# Hospitals and the Spirit Tax

**Published:** 1915-07-03

**Authors:** 


### HOSPITALS AND THE SPIRIT TAX.

The proposed new clause providing for the use of duty-free spirit in hospitals for the manufacture of tinctures, &c., was not inserted in the Finance Bill on the Committee stage in the House of Commons on Tuesday night. Opposition to the clause came from quarters from which it was least expected—namely, from the British Medical Association and the Pharmaceutical Society. All the arguments urged against the clause could have been met without difficulty, but, unfortunately, there seemed to be nobody in the House who was prepared at the moment to support the case for the hospitals in such a thorough way as the case against the use of duty-free spirit was presented. This was probably because the line taken by the opponents had not been anticipated. There is still the frail hope that the clause will receive more support when it is brought up again on the report stage, but the prospects are much less favourable than they appeared to be.

Elsewhere in this issue of The Hospital appears a letter from a correspondent, who deals with the definition of "public hospital" embodied in this unfortunate clause. The suggestion in the article to which he alludes—namely, that some critics would think that the somewhat wide definition might open the door to abuse—was certainly well founded, as a glance at the proposed amendments printed in Tuesday's Parliamentary papers will show. It will be seen that no fewer than five members had given notice to move the deletion from the definition of the words "or partly," while three members had given notice to move the deletion of the word "by" preceding the words "voluntary subscriptions." Had these amendments been adopted the definition would have read: "The expression 'public hospital' means a hospital supported by any public authority or wholly out of any public or charitable funds or voluntary subscriptions." Whatever may have been the intention of the framers of the clause, the view was undoubtedly held that the word "partly" governed not only "out of public or charitable funds," but "by voluntary subscriptions." In any case had the clause been passed the Commissioners of Customs and Excise would have had very wide powers of discrimination. The clause provided that the Commissioners "may authorise a person to receive spirits without payment of duty," and it also required the treasurers of the hospital to show "to the satisfaction" of the Commissioners that certain things had been done. Had the clause passed into law the Commissioners would have made regulations thereunder, and, as was intimated in the article referred to, it would have been very difficult for a bogus hospital to benefit by a concession that was intended to apply only to *bona-fide* institutions.

In matters of this kind wide discretionary powers must be vested in the Commissioners. Unfortunately, however, the House has not even endorsed the principle embodied in the proposed new clause, and under these circumstances the question of details is not one of the highest importance.

